# Dental Outcomes After Medicaid Insurance Coverage Expansion Under the Affordable Care Act

**DOI:** 10.1001/jamanetworkopen.2021.24144

**Published:** 2021-09-30

**Authors:** Hawazin W. Elani, Ichiro Kawachi, Benjamin D. Sommers

**Affiliations:** 1 Department of Oral Health Policy and Epidemiology, Harvard School of Dental Medicine, Boston, Massachusetts; 2 Department of Health Policy and Management, Harvard T.H. Chan School of Public Health, Boston, Massachusetts; 3 Department of Social and Behavioral Sciences, Harvard T.H. Chan School of Public Health. Boston, Massachusetts; 4 Department of Medicine, Brigham and Women’s Hospital, Harvard Medical School, Boston, Massachusetts

## Abstract

**Importance:**

Dental coverage for adults is a state option in Medicaid, and despite significant gains in coverage after the Medicaid expansion under the Affordable Care Act (ACA), dental outcomes among adults in expansion states remain unexplored.

**Objective:**

To explore the association of state coverage of dental benefits through Medicaid expansion with clinical dental outcomes.

**Design, Setting, and Participants:**

This cross-sectional study analyzed data from the National Health and Nutrition Examination Survey from 2009 to 2018. Included participants were low-income adults aged 19 to 64 years with income up to 138% of the federal poverty level. The study used a difference-in-differences analysis to compare changes from before to after ACA expansion in expansion states vs in control states. Changes were examined in the full sample and separately in states that did and did not provide Medicaid adult dental benefits. We defined a state as providing Medicaid adult dental benefits if it covered services beyond emergency dental benefits in 2014. Data were analyzed from November 2020 to March 2021.

**Exposures:**

Medicaid expansion under the ACA.

**Main Outcomes and Measures:**

Rates of health coverage, having a dental visit, affordability of dental care in the past year, poor oral health, and teeth flossing were obtained from self-reported data. Mean number of missing teeth and prevalence of untreated decayed teeth, filled teeth, and functional dentition were obtained from clinical examination data.

**Results:**

Among 7637 low-income adults, the mean (SD) age was 37.8 (13.4) years and 4153 (weighted percentage, 54.5 %) were women. At baseline, 1732 low-income adults in nonexpansion states compared with 2520 low-income adults in expansion states were more likely, as shown by weighted percentage, to be Black (473 individuals [21.0%] vs 508 individuals [15.1%]) and US born (1281 individuals [76.7%] vs 1613 individuals [69.6%]). In the full sample, Medicaid expansion, compared with nonexpansion, was associated with an increased rate of seeing a dentist in the prior year (12.4 percentage points; 95% CI 4.6 to 20.2 percentage points; *P* = .003). In expansion states that provided dental benefits, compared with nonexpansion states that provided dental benefits, the expansion was associated with increases in rates of Medicaid coverage (8.2 percentage points; 95%CI 0.5 to 15.8 percentage points; *P* = .04) and having seen a dentist in the previous year (11.4 percentage points, 95% CI, 3.7 to 19.1 percentage points; *P* = .006) and decreases in the uninsured rate (−12.6 percentage points, 95% CI −18.9 to −6.4 percentage points; *P* < .001) and prevalence of untreated decayed teeth (−16.8 percentage points; 95% CI, −25.5 to −8.0 percentage points; *P* = .001). In states without Medicaid dental benefits, the expansion was associated with an increase in the mean number of missing teeth (1.3 teeth; 95% CI 0.1 to 2.5 percentage points; *P* = .04) and a decrease in the prevalence of functional dentition (−8.7 percentage points; 95% CI, −14.1 to −3.3 percentage points; *P* = .003) compared with nonexpansion states.

**Conclusions and Relevance:**

This study found that the combination of Medicaid expansion and coverage of Medicaid dental benefits was associated with improved oral health among low-income adults.

## Key Points

**Question:**

Is dental insurance through Medicaid expansion under the Affordable Care Act (ACA) associated with improved oral health outcomes among low-income adults?

**Findings:**

In this cross-sectional study of 7637 low-income adults, the ACA Medicaid expansion in states that expanded Medicaid and offered dental coverage, compared with nonexpansion states, was associated with improved health coverage, increased access to dental care, decreased prevalence of untreated decayed teeth, and improved oral health–related behaviors (ie, flossing). In states without dental coverage, the expansion was associated with an increase in the mean number of missing teeth and a decrease in the prevalence of functional dentition among low-income adults compared with nonexpansion states.

**Meaning:**

This study found that the combination of Medicaid expansion and coverage of Medicaid dental benefits was associated with improved coverage and access to dental care among low-income adults and with improvements in clinical indicators associated with oral health.

## Teaser

This cross-sectional study compares dental outcomes among low-income adults in states that expanded Medicaid coverage under the Affordable Care Act (ACA) compared with those in states that did not expand Medicaid coverage.

## Introduction

Persistent disparities in oral health pose a major public health challenge given that low-income and racial and ethnic minority populations continue to experience a greater burden of dental disease.^1,2^ There have been significant gains in dental coverage since the implementation of the Affordable Care Act (ACA).^3,4^ However, expanding coverage alone may not be associated with sufficiently improved use of dental services or oral health status. Prior studies have primarily focused on examining changes in dental coverage and access to dental care,^5,6,7^ but the association of the ACA Medicaid expansion with clinical dental outcomes remain unexplored, to our knowledge.

Adult dental coverage in Medicaid remains a state optional benefit, and 31 states and the District of Columbia offered dental services to Medicaid nonelderly adults beyond emergency services as of 2020.^8,9^ In the current context of evolving Medicaid policy at the federal and state level and as states consider strategies to cut Medicaid spending, the question of the association of policy changes with oral health outcomes and disparities remains essential.

In this study, we used data from the National Health and Nutrition Examination Survey (NHANES).^10^ This is a unique data source in that it is the only national survey in the US that contains detailed clinical dental examination data, allowing the examination of clinical dental outcomes, rather than just patient-reported dental outcomes, which are often confounded by access to care. To our knowledge, there have been no studies evaluating the association of the ACA expansion with unmet dental need outcomes using clinical measures. A 2020 study^11^ using self-reported data from the Medical Expenditure Panel Survey 2 years after the expansion found that the ACA was associated with improved access to dental treatments. Thus, the objective of our study was to examine the association of the Medicaid expansion with dental outcomes and use of dental services among low-income adults and to investigate whether changes in dental outcomes varied by states’ coverage of dental benefits. Following previous research, we defined a state as providing Medicaid adult dental benefits if it covered services beyond emergency dental benefits in 2014.^3,5,6^

## Methods

This cross-sectional study used deidentified data and was determined to not be human subjects research by the institutional review board of the Harvard Faculty of Medicine and thus exempt from review and written informed consent. We followed the Strengthening the Reporting of Observational Studies in Epidemiology (STROBE) reporting guideline.

### Study Design

We used a difference-in-differences linear regression analysis to compare changes in outcomes from before to after Medicaid expansion under the ACA in expansion states vs in control states. Our study period included 5 years before and after the policy implementation in 2014. We examined changes in outcomes in the full sample and then separately in states that did and did not provide Medicaid adult dental benefits to investigate whether changes varied by states’ coverage of dental benefits. In all analyses, we compared expansion states with nonexpansion states. For example, when we restricted the analysis to states providing Medicaid adult dental coverage, we compared expansion states with dental coverage with nonexpansion states with dental coverage.

### Data

We analyzed data from the NHANES that is conducted annually by the National Center for Health Statistics.^10^ The NHANES used a stratified, multistage probability-sampling design and included a nationally representative sample of the noninstitutionalized population of the US. The survey included information about participants’ demographic characteristics, insurance coverage status, and health conditions, as well as clinical examination data. The oral health component comprised household interviews followed by dental examination conducted by trained dentists at a mobile examination center.^12^ We used NHANES data from 2009 to 2018 and obtained access to restricted state identifiers through Federal Statistical Research Data Centers. These centers provided us with deidentified state variables as well as indicator variables for each state’s expansion and dental coverage status.

### Measures

The study outcomes included 4 domains: health coverage, access to dental care, clinically determined dental outcomes, and self-reported dental outcomes. Health coverage outcomes were Medicaid coverage and uninsured status during the previous 12 months. Being uninsured was defined as having no health insurance purchased directly or obtained through employment or any government programs, such as Medicare or Medicaid. Outcomes for access to dental care included seeing a dentist in the previous year and inability to afford dental care in the previous year. Data on access to dental care measures were available for NHANES survey years 2011 to 2018.

Clinical measures included number of missing teeth and the presence of untreated decayed teeth, filled teeth, and functional dentition; these were expressed for the population as mean number of missing teeth and prevalence of untreated decayed teeth, filled teeth, and functional dentition. The presence of functional dentition was defined as having at least 20 teeth and is a widely used indicator for assessing whether an individual has adequate dentition to maintain chewing ability.^13,14^ Self-reported dental outcomes were self-rated oral health (which was categorized into 2 groups: excellent, very good, or good vs fair or poor) and oral health–related behavior, which was assessed by whether each respondent reported using dental floss at least once a day. All outcomes were binary variables except for the number of missing teeth, which ranged from zero to 32 teeth per individual.

### Statistical Analysis

We estimated a linear regression model to assess the association of Medicaid expansion with each outcome^15^ using the following linear probability model:

*Y_ist_ = β_0_ + ****β****_1_ year + ****β****_2_ state + β_3_ (expansion state × post 2014) _st_ + ****X****_ist_ + ****Z****_ist_ + ε_ist._*

*Y_ist_* represents the oral health outcome of interest. The subscripts *i*, *s*, and *t* index individual, state, and time, respectively. **X***_ist_* represents individual-level covariates, **Z***_ist_* represents state-level covariates, and ε*_ist_* is the error term. ***β****_1_*and ***β****_2_* are vectors of time and state fixed effects, respectively. Year fixed effects control for any secular trends in the outcome that are common across states. State fixed effects control for any unmeasured differences between states. β_3_ is the difference-in-differences estimate that captures the mean difference for each outcome from before to after the Medicaid expansion in expansion vs in control states.

We restricted the sample to adults ages 19 to 64 years with income up to 138% of the federal poverty level. All models were adjusted for age, sex, race and ethnicity, education, marital status, employment, citizenship status, unemployment rate per state by year, number of dentists per capita in each state by year, and state fixed effects. Participants responded to NHANES questions and self-identified their race and ethnicity. The variable for race and ethnicity was included as a covariate in the model (ie, for adjustment). All analyses used robust standard errors clustered by state to account for serial autocorrelation.^16^

In our primary analysis, we defined states as expansion states if they implemented Medicaid expansion at any time during our study period (eTable 1 in the Supplement). Because some pregnant women have different eligibility requirements for Medicaid and to account for early improvement in states before the implementation of the ACA, we conducted a sensitivity analysis that excluded pregnant women and states that implemented coverage expansion prior to 2014. To test the assumption of our difference-in-differences design, we examined whether trends in outcomes in expansion vs in control states were parallel before the Medicaid expansion. We first examined yearly trends visually, then conducted a placebo expansion test to examine whether trends in expansion and nonexpansion states differed in the preexpansion period. For the placebo test, we used a placebo difference-in-differences model and estimated equation 1 but using NHANES data from 2009 to 2013 and year 2012 as the ACA placebo implementation year. Because the NHANES is a 2-year cycle survey, we used year 2012 rather than 2013 as the placebo expansion year so that we had 2 years after the placebo ACA implementation to ensure that we had adequate power to detect changes.

We used NHANES survey weights to account for the complex survey design. We used Stata statistical software version 15.1 for all analyses. Statistical significance was based on 2-sided *P* value ≤ .05. Data were analyzed from November 2020 to March 2021.

## Results

Among 7637 low-income adults, the mean (SD) age was 37.8 (13.4) years and 4153 (weighted percentage, 54.5%) were women. Baseline characteristics of study sample are presented in Table 1. At baseline, 1732 low-income adults in nonexpansion states compared with 2520 low-income adults in expansion states were more likely, as shown by weighted percentage, to be US citizens (82.4% vs 78.8%), US born (1281 individuals [76.7%] vs 1613 individuals [69.6%]), married (50.7% vs 48.9%), and Black (473 individuals [21.0%] vs 508 individuals [15.1%]). Individuals in nonexpansion states were more likely, as shown by weighted percentage, to be White (661 individuals [48.3%] vs 881 individuals [47.7%]) and less likely to be of other racial backgrounds (121 individuals [6.5%] vs 277 individuals [7.8%]) or to be Mexican American (298 individuals [14.8%] vs 569 individuals [19.7%]) or of other Hispanic origin (179 individuals [9.4%] vs 285 individuals [9.9%]).

**Table 1.** Characteristics of Low-Income Adults in Medicaid Expansion and Control States at Baseline

Characteristic^a^	Participants, No. (weighted %)^b^	
	In expansion states (n = 2520)	In nonexpansion states (n = 1732)
Sex		
Men	1163 (45.9)	800 (46.9)
Women	1357 (54.1)	932 (53.1)
Age, y^c^		
19-34	1057 (46.0)	765 (47.8)
35-44	524 (21.3)	351 (20.9)
45-54	506 (19.8)	313 (17.1)
55-64	433 (12.9)	303 (14.2)
Race and ethnicity		
Mexican American	569 (19.7)	298 (14.8)
Other Hispanic	285 (9.9)	179 (9.4)
Non-Hispanic		
White	881 (47.7)	661 (48.3)
Black	508 (15.1)	473 (21.0)
Other race or ethnicity^d^	277 (7.8)	121 (6.5)
Education		
<High school	915 (34.1)	541 (30.7)
High school graduate	611 (26.0)	453 (27.6)
>High school	865 (39.8)	645 (41.6)
Marital status		
Married	1232 (48.9)	806 (50.7)
Single	1161 (51.1)	835 (49.3)
Citizenship		
US citizen	1914 (78.8)	1399 (82.4)
Foreign citizen	597 (20.9)	330 (17.4)
Nativity		
US born	1613 (69.6)	1281 (76.7)
Foreign born	905 (30.4)	451 (23.3)
Household income, % of federal poverty level^e^		
<0.5	585 (23.6)	387 (20.6)
0.5-1.0	1112 (43.9)	706 (41.0)
>1.0	823 (32.4)	639 (38.4)

^a^ Data are from National Health and Nutrition Examination Survey years 2009 to 2013.

^b^ Expansion states can include Alaska, Arkansas, Arizona, California, Colorado, Connecticut, the District of Columbia, Delaware, Hawaii, Iowa, Illinois, Indiana, Kentucky, Louisiana, Maryland, Massachusetts, Michigan, Minnesota, Montana, North Dakota, New Hampshire, New Jersey, New Mexico, Nevada, New York, Ohio, Oregon, Pennsylvania, Rhode Island, Vermont, Washington, and West Virginia. Nonexpansion states can include Alabama, Florida, Georgia, Idaho, Kansas, Maine, Missouri, Mississippi, North Carolina, Nebraska, Oklahoma, South Carolina, South Dakota, Tennessee, Texas, Utah, Virginia, Wisconsin, and Wyoming. Percentages may not add to 100 because of rounding.

^c^ Study sample limited to adults ages 19 to 64 years.

^d^ Includes non-Hispanic Asian and multiracial individuals.

^e^ Low-income sample limited to adults with income up to 138% of the federal poverty level.

### Trends in Outcomes

**Figure 1.**
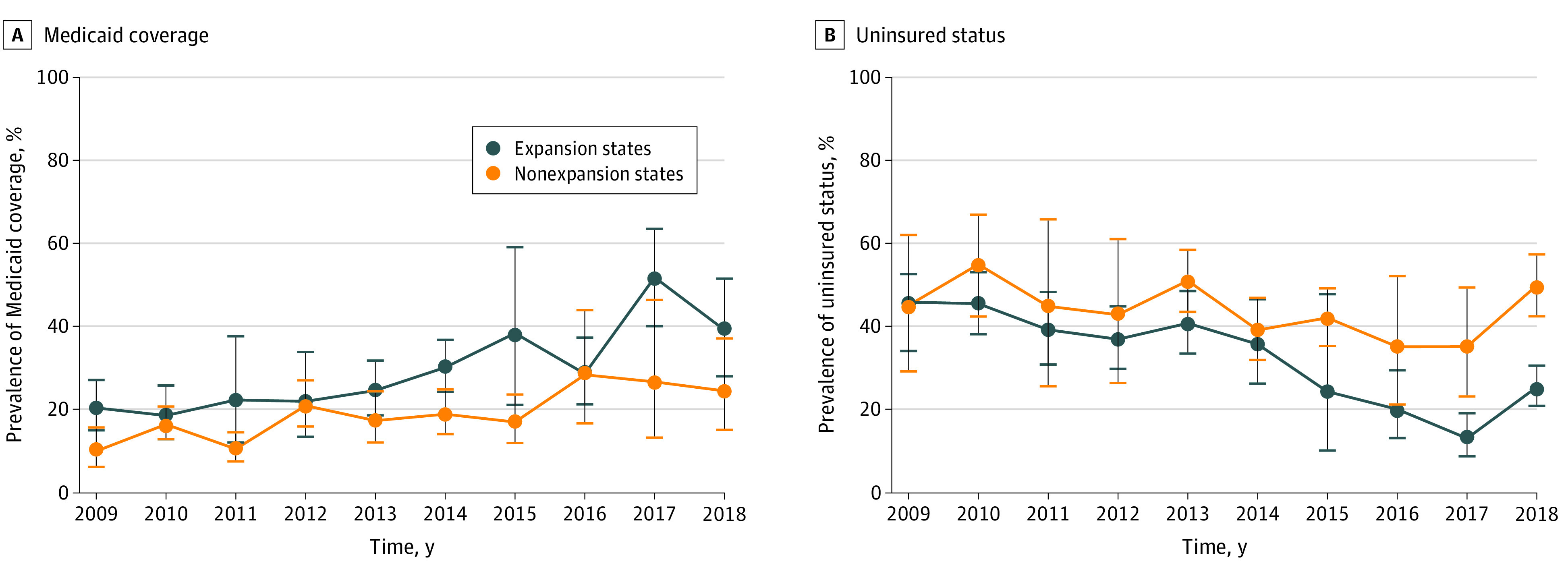
Unadjusted Trends in Health Coverage Outcomes by Medicaid Expansion Status and Survey Year

**Figure 2.**
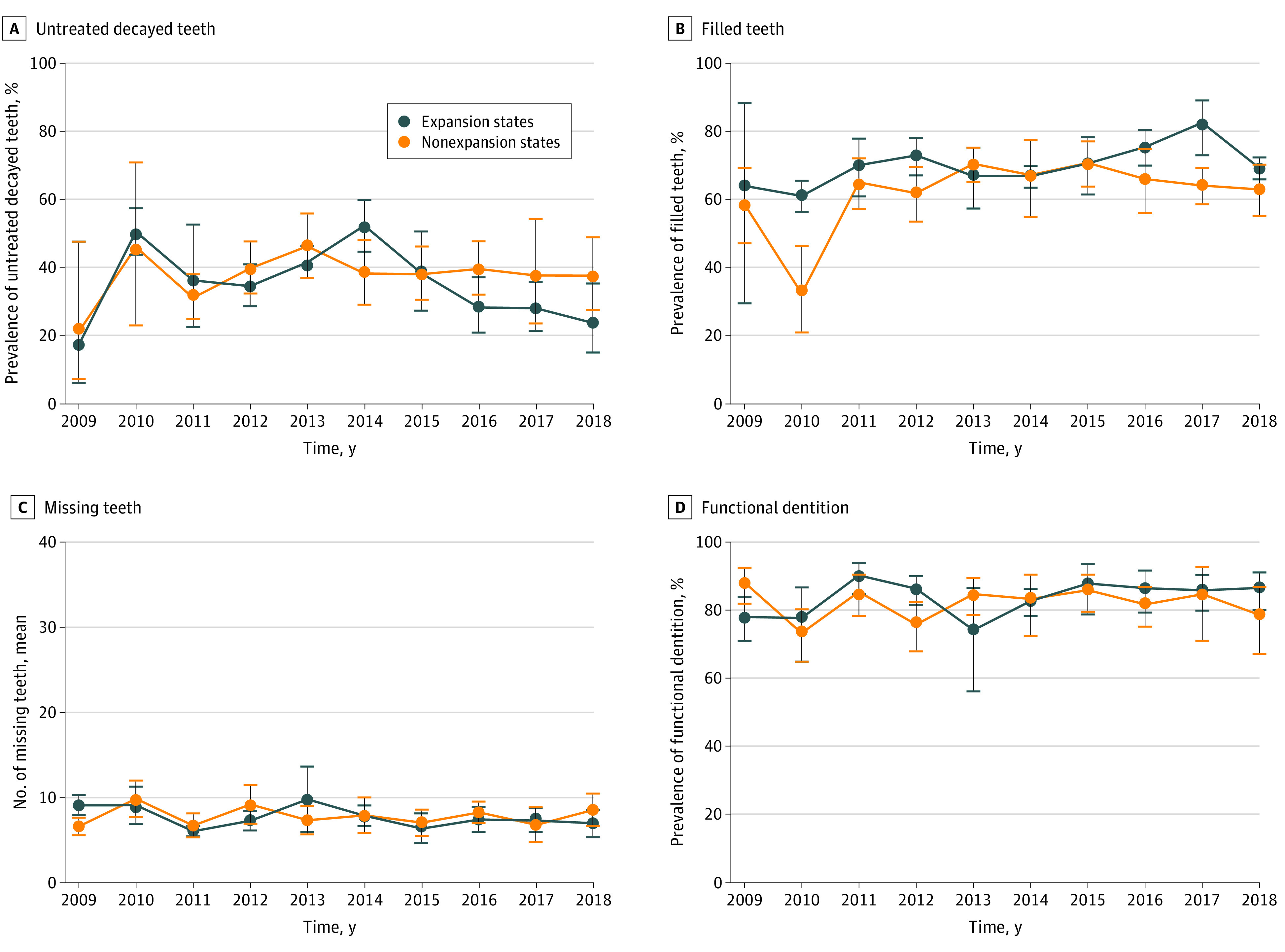
Unadjusted Trends in Clinically Examined Dental Outcomes by Medicaid Expansion Status and Survey Year

Figure 1, 2, and 3 present unadjusted trends for each outcome in expansion and nonexpansion states according to NHANES survey year. Most outcomes demonstrated improved trends after the Medicaid expansion. Post-ACA, compared with control states, expansion states had larger increases in rates of Medicaid coverage, seeing a dentist in the past year, and filled teeth and greater decreases in rates of uninsured status, inability to afford to dental care, and untreated decayed teeth.

**Figure 3.**
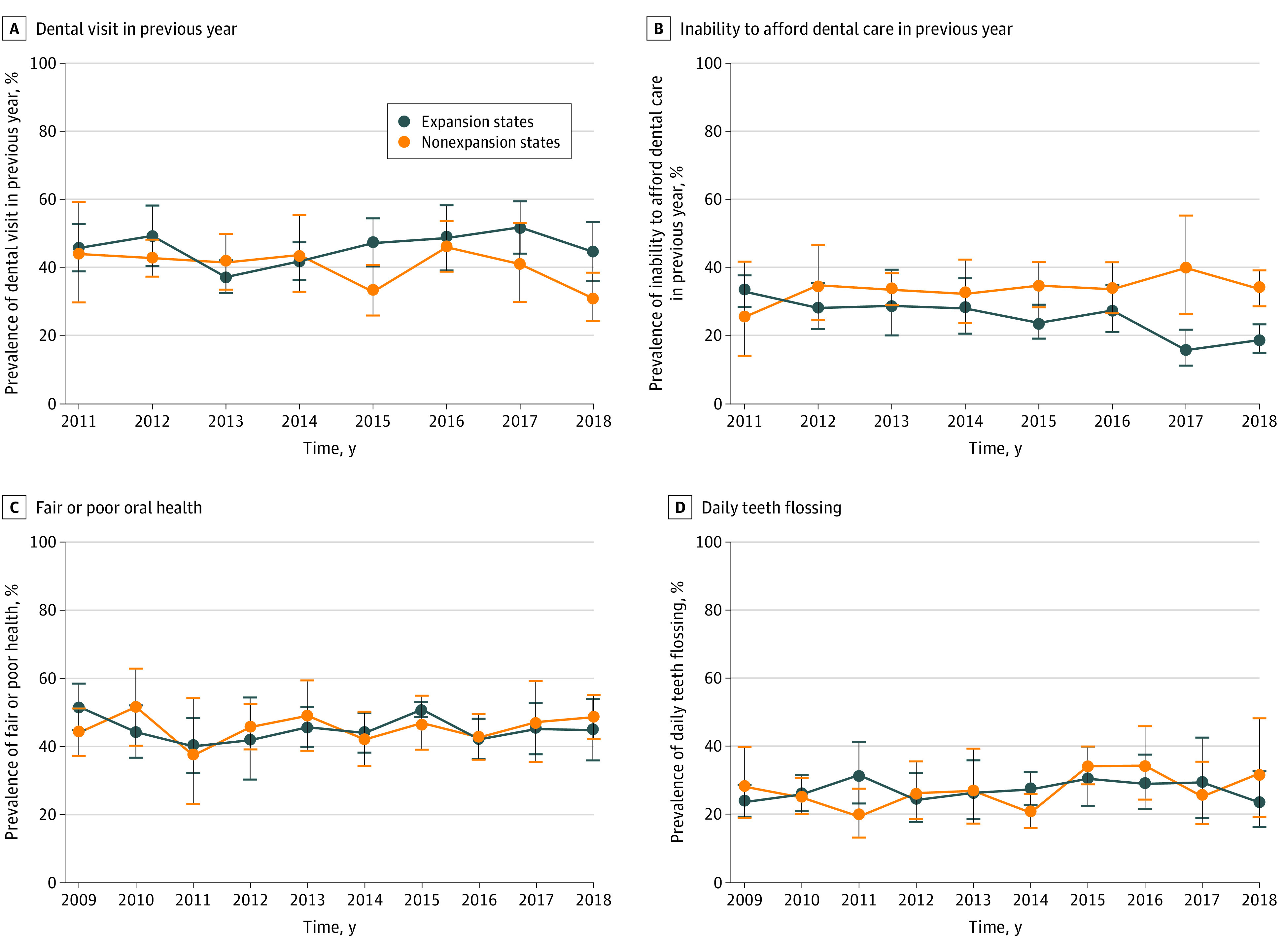
Unadjusted Trends in Self-reported Dental Outcomes by Medicaid Expansion Status and Survey Year

### Changes in Health Coverage and Access to Dental Care

Results from difference-in-differences analysis are presented in Table 2. In the full sample, the expansion was not associated with changes in health coverage outcomes. However, in expansion states with dental benefits, the expansion was associated with an increase in Medicaid coverage of 8.2 percentage points (95% CI, 0.5 to 15.8 percentage points; *P* = .04) and a decrease of 12.6 percentage points in the uninsured rate (95% CI, −18.9 to −6.4; *P* < .001) compared with nonexpansion states with dental benefits.

**Table 2.** Changes in Outcomes Among Low-Income Adults After State Medicaid Expansion in Full Sample and by State Dental Benefits

Outcome^a^	Unadjusted proportion before ACA, % (95% CI)		Difference-in-differences: net change after expansion	
	Medicaid expansion states^b^	Nonexpansion states^c^	Percentage points (95% CI)	*P* value
**Health coverage**				
Medicaid coverage				
Full sample	21.8 (18.2 to 25.9)	14.7 (11.7 to 18.1)	5.1 (−2.0 to 12.2)	.15
States with dental benefits	22.9 (17.8 to 29.0)	17.7 (13.2 to 23.3)	8.2 (0.5 to 15.8)	.04
States without dental benefits	20.0 (15.4 to 25.6)	13.3 (10.5 to 16.6)	2.8 (−13.0 to 18.7)	.71
Uninsured status				
Full sample	41.3 (38.2 to 44.6)	47.5 (42.0 to 53.0)	−6.2 (−16.2 to 3.7)	.21
States with dental benefits	37.6 (33.5 to 41.9)	41.3 (29.2 to 54.5)	−12.6 (−18.9 to −6.4)	<.001
States without dental benefits	47.2 (43.1 to 51.3)	50.3 (39.2 to 61.4)	−2.6 (−17.9 to 12.6)	.72
**Access to dental care**				
Dental visit in previous year				
Full sample	43.9 (38.9 to 49.0)	42.8 (36.6 to 49.2)	12.4 (4.6 to 20.2)	.003
States with dental benefits	47.2 (40.0 to 54.5)	42.4 (34.3 to 50.9)	11.4 (3.7 to 19.1)	.006
States without dental benefits	38.0 (31.9 to 44.6)	42.9 (34.5 to 51.6)	23.0 (3.5 to 42.6)	.02
Inability to afford dental care in the previous year				
Full sample	29.5 (25.7 to 33.5)	30.2 (23.9 to 37.4)	−10.2 (−22.3 to 1.8)	.09
States with dental benefits	27.4 (22.4 to 33.0)	27.5 (22.9 to 32.5)	−19.3 (−29.0 to −9.6)	.001
States without dental benefits	33.2(27.5 to 39.5)	30.9 (21.9 to 41.6)	−12.7 (−31.2 to 5.9)	.17
**Clinically examined dental outcomes**				
Untreated decayed teeth				
Full sample	37.7 (33.9 to 41.7)	37.5 (31.6 to 43.9)	−5.0 (−14.7 to 4.7)	.30
States with dental benefits	36.2 (30.8 to 42.0)	37.2 (30.4 to 44.7)	−16.8 (−25.5 to −8.0)	.001
States without dental benefits	40.2 (31.6 to 49.5)	37.6 (30.1 to 45.8)	−2.7 (−15.0 to 9.7)	.66
Filled teeth				
Full sample	69.8 (65.2 to 74.1)	64.9 (60.6 to 69.0)	3.1 (−7.0 to 13.2)	.54
States with dental benefits	68.9 (62.1 to 74.9)	60.9 (51.7 to 69.4)	11.2 (−1.1 to 23.5)	.07
States without dental benefits	71.4 (65.7 to 76.6)	65.9 (60.7 to 70.8)	−0.9 (−18.3 to16.5)	.91
No. of missing teeth, mean (95% CI)^d^				
Full sample	8.2 (6.9 to 9.5)	7.9 (6.5 to 9.2)	−0.4 (−1.7 to 0.8)	.49
States with dental benefits	9.1 (7.3 to 11.0)	10.0 (8.3 to 11.7)	−1.2 (−3.0 to 0.5)	.16
States without dental benefits	6.7 (6.0 to 7.5)	7.1 (6.2 to 7.9)	1.3 (0.1 to 2.5)	.04
Functional dentition				
Full sample	81.6 (75.6 to 86.4)	81.6 (76.1 to 86.1)	2.0 (−4.1 to 8.1)	.51
States with dental benefits	78.2 (69.0 to 85.2)	73.9 (67.0 to 79.7)	5.7 (−1.9 to 13.3)	.13
States without dental benefits	87.1 (82.8 to 90.4)	84.5 (81.1 to 87.4)	−8.7 (−14.1 to −3.3)	.003
**Self-reported dental outcomes**				
Fair or poor oral health				
Full sample	44.0 (39.7 to 48.3)	44.4 (36.4 to 52.6)	−2.3 (−9.4 to 4.8)	.51
States with dental benefits	42.8 (36.5 to 49.2)	49.5 (42.1 to 56.9)	−1.3 (−14.9 to 12.3)	.85
States without dental benefits	45.9 (41.9 to 50.0)	42.4 (33.7 to 51.5)	−1.6 (−17.7 to 14.5)	.84
Daily teeth flossing				
Full sample	26.3 (22.7 to 30.2)	24.6 (21.3 to 28.3)	0.3 (−9.2 to 9.8)	.95
States with dental benefits	22.5 (18.9 to 26.7)	24.7 (19.9 to 30.1)	10.8 (4.3 to 17.3)	.002
States without dental benefits	32.1 (27.9 to 36.6)	24.6 (20.3 to 29.5)	−13.4 (−23.5 to −3.3)	.01

Abbreviation: ACA, Affordable Care Act.

^a^ Data are from National Health and Nutrition Examination Survey years 2009 to 2018. Study sample limited to adults ages 19 to 64 years with income up to 138% of the federal poverty level. Models adjusted for age, sex, race and ethnicity, education, marital status, employment, citizenship, unemployment rate per state by year, number of dentists per capita in each state per year, and state. All analyses used robust standard errors clustered by state.

^b^ Expansion states can include Alaska, Arkansas, Arizona, California, Colorado, Connecticut, the District of Columbia, Delaware, Hawaii, Iowa, Illinois, Indiana, Kentucky, Louisiana, Massachusetts, Maryland, Michigan, Minnesota, Montana, North Dakota, New Hampshire, New Jersey, New Mexico, Nevada, New York, Ohio, Oregon, Pennsylvania, Rhode Island, Vermont, Washington, and West Virginia. Expansion states that provided dental benefits can include Alaska, Arkansas, California, Colorado, Connecticut, the District of Columbia, Iowa, Illinois, Indiana, Kentucky, Massachusetts, Michigan, Minnesota, North Dakota, New Jersey, New Mexico, New York, Ohio, Oregon, Pennsylvania, Rhode Island, Vermont, and Washington. Expansion states that did not provide dental benefits can include Arizona, Delaware, Hawaii, Louisiana, Maryland, Montana, New Hampshire, Nevada, and West Virginia.

^c^ Nonexpansion states can include Alabama, Florida, Georgia, Idaho, Kansas, Maine, Missouri, Mississippi, North Carolina, Nebraska, Oklahoma, South Carolina, South Dakota, Tennessee, Texas, Utah, Virginia, Wisconsin, and Wyoming. Nonexpansion states that provided dental benefits can include North Carolina, Nebraska, South Carolina, South Dakota, Wisconsin, and Wyoming. Nonexpansion states that did not provide dental benefits can include Alabama, Florida, Georgia, Idaho, Kansas, Maine, Missouri, Mississippi, Oklahoma, Tennessee, Texas, Utah, and Virginia.

^d^ The difference-in-differences net change for missing teeth is expressed as No. of missing teeth (ie, the difference between means of No. of missing teeth).

For access to dental care outcomes, the expansion was associated with an increase in the proportion of low-income adults reporting seeing a dentist in the previous year in the full sample (12.4 percentage points; 95% CI, 4.6 to 20.2 percentage points; *P* = .003), in states with dental benefits (11.4 percentage points; 95% CI, 3.7 to 19.1 percentage points; *P* = .006), and in states without dental benefits (23.0 percentage points; 95% CI, 3.5 to 42.6 percentage points; *P* = .02). The expansion was also associated with a decrease in the inability to afford dental care in states providing dental benefits (−19.3 percentage points; 95% CI, −29.0 to −9.6; *P* = .001).

### Changes in Dental Outcomes

Medicaid expansion, compared with no expansion, was associated with a decrease in the prevalence of untreated decayed teeth in states providing dental benefits (−16.8 percentage points; 95% CI, −25.5 to −8.0; *P* = .001), but there were no statistically significant changes in the prevalence of filled teeth. In states without dental benefits, the expansion was associated with an increase in the mean number of missing teeth (1.3 teeth; 95% CI, 0.1 to 2.5 percentage points; *P* = .04) and a decrease in the prevalence of functional dentition (−8.7 percentage points; 95% CI, −14.1 to −3.3 percentage points; *P* = .003) in expansion states compared with nonexpansion states.

The expansion was not associated with changes in the proportion of low-income adults reporting fair or poor oral health. However, Medicaid expansion was associated with an increase in the proportion of low-income adults reporting flossing their teeth daily in states providing dental benefits (10.8 percentage points; 95% CI, 4.3 to 17.3 percentage points; *P* = .002) and a decrease in the proportion reporting flossing in states without dental benefits (−13.4 percentage points; 95% CI, −23.5 to −3.3 percentage points; *P* = .01).

### Sensitivity Analyses

Results from models excluding pregnant women were similar to those in our main analysis (eTable 2 in the Supplement). When we excluded states that expanded Medicaid prior to 2014, the results were similar. In the full sample for this analysis, the expansion was associated with an increase in Medicaid coverage and a decrease in the inability to afford dental care, but changes in seeing a dentist in the previous year were not statistically significant. In this analysis, there were statistically significant increases in the prevalence of filled teeth in the full sample and in states providing dental benefits, whereas in states without dental benefits, changes in the mean number of missing teeth were no longer significant.

In the placebo test, there were no statistically significant changes for most outcomes, providing support for our difference-in-differences design. However, the prevalence of being uninsured, having a dental visit, and being able to afford dental care in the previous year had statistically significant placebo coefficients (eTable 3 in the Supplement).

## Discussion

Using nationally representative data from 2009 to 2018, this cross-sectional study assessed the association of the ACA expansion with health coverage, access to dental care, and clinical dental outcomes by states’ coverage of adult dental benefits among low-income adults. We found that in states that expanded Medicaid and offered dental coverage compared with nonexpansion states, the ACA Medicaid expansion was associated with improved health coverage, increased access to dental care, decreased prevalence of untreated decayed teeth, and improved oral health–related behaviors (ie, increased prevalence of flossing). By contrast, in states without dental coverage, the expansion was associated with an increase in teeth loss (ie, an increase in the mean number of missing teeth and a decrease in the prevalence of functional dentition). Overall, our analysis provides evidence that coverage of dental services is associated with improved dental care and oral health for the low-income population.

Our analysis provides new evidence about the long-term association of the Medicaid expansion with oral health outcomes using objective clinical indicators. We found that in states that expanded Medicaid coverage and provided adult dental benefits, the expansion was associated with decreased prevalence of untreated decayed teeth among low-income adults. In contrast, we found that in expansion states without adult dental benefits, the expansion was associated with increases in the loss of teeth and subsequently a decrease in the prevalence of functional dentition, which could be associated with decreased chewing ability and overall health among individuals. Most dental diseases are preventable and can be treated at a low cost if diagnosed at an early enough stage to prevent teeth loss. Our findings thus underscore the association of early access to dental care with improved oral health, particularly for low-income populations. In states providing dental benefits, the expansion was associated with improved access to dental treatments and affordability of these treatments, such as crowns and root canal therapy, which was associated with restored oral health and maintenance of natural dentition among beneficiaries, compared with states without dental benefits, where extraction of teeth may be the only affordable dental treatment. For example, the mean cost for a molar tooth extraction by a general dentist is approximately $170, compared with $2155 for a root canal treatment and a crown.^17^

Our findings on changes in health coverage are consistent with prior analyses of the Medicaid expansion,^18,19,20,21^ although we found significant increases in Medicaid coverage and decreases in uninsured rates only in states that expanded coverage and provided adult dental benefits. However, the point estimates for Medicaid coverage and uninsured status for the full sample in our study are in the expected direction, with an increase of 5.1 percentage points in Medicaid coverage and a decrease of 6.2 percentage points in uninsured rates, although these estimates had wide CIs and were not statistically significant. The NHANES does not necessarily visit all states in each survey cycle, and thus the number of states in the NHANES is smaller than in other federal surveys, which may be associated with decreased precision. Our results are also consistent with prior research finding that the expansion was associated with increases in the proportion of low-income adults reporting having seen a dentist in the previous year.^3,6,7^ Although our point estimates are larger than those found in previous research, our 95% CIs included estimates from those studies. We also found that the expansion was associated with improved affordability of dental care in states providing Medicaid dental benefits. However, it is important to note that those increases were also statistically significant in our placebo test, suggesting that these changes were associated with differences in preexpansion trends between expansion and nonexpansion states.

This study also offers new evidence on how the Medicaid expansion may be associated with oral health–related behaviors. We found significant increases in rates of daily flossing after the expansion in states providing dental benefits and significant decreases in states without dental benefits. These differences suggest that coverage gains after the ACA expansion may have enabled low-income adults to access dental care, which may have been associated with adoption of preventive behaviors, such as flossing.

Although our findings suggest that the expansion was associated with increases in access to dental services and use of those services, we did not find statistically significant improvements in self-rated oral health. These findings may be associated with increasing awareness of unmet dental needs among Medicaid enrollees. Self-rated oral health is a subjective measure of oral health status. Therefore, it is possible that coverage gains were associated with increased rates of seeking dental care among beneficiaries, but because of prolonged lack of access to dental services, a large proportion of beneficiaries may have poor dental conditions, which may be associated with dissatisfaction with their oral health. Additionally, being diagnosed by a dentist may be associated with improved accuracy in assessing oral health among beneficiaries. Furthermore, cost remains a significant barrier to use of dental services, and Medicaid dental coverage varies widely across states. Thus, particularly in states providing limited dental coverage, some Medicaid enrollees may be unable to afford the out-of-pocket cost for their dental treatment.^22^

### Limitations

This study has several limitations. We used restricted data from the NHANES, and our data user agreement precluded us from identifying individual states or analyzing small groups of states. Therefore, we were unable to separately analyze states that expanded Medicaid after 2014 (ie, Alaska, Indiana, Louisiana, Montana, and Pennsylvania) and we included these states in the expansion group, which may be associated with misclassification of some states’ treatment assignments. In addition, self-reported outcomes are susceptible to recall bias; however, this likely affected expansion and control states. Additionally, our difference-in-differences design provides mean changes in outcomes among all low-income adults after the expansion. Evidence from prior studies suggests that Medicaid expansion may be associated with different outcomes in access to dental services among minority populations compared with nonminority populations.^3,7^ However, owing to limited statistical power, we were unable to conduct subgroup analyses to examine racial or ethnic disparities in the receipt of dental care.

## Conclusions

Although adult dental benefits remain optional in Medicaid, we found that the combination of Medicaid expansion and coverage of adult dental benefits was associated with improved oral health among low-income adults. This study’s findings suggest that the ACA expansion was associated with not only improved coverage and access to dental care, but also improvements in clinical indicators of oral health. Low-income adults in states that provided more generous adult dental benefits in Medicaid had significant improvements in dental health. These findings suggest that improved access to dental care is associated with health benefits and may guide policy makers aiming to implement robust Medicaid programs.

## Notes

Supplement. **eTable 1.** Classification of States by Medicaid Expansion and State Dental Benefits Status **eTable 2.** Sensitivity Analyses **eTable 3.** Robustness Checks
