# Supplementary material for: Dental Outcomes After Medicaid Insurance Coverage Expansion Under the Affordable Care Act
Source: JAMA Netw Open. 2021 Sep 30;4(9):e2124144. doi: 10.1001/jamanetworkopen.2021.24144 (PMC8485174; doi:10.1001/jamanetworkopen.2021.24144)
Supplement: Supplement. — eTable 1. Classification of States by Medicaid Expansion and State Dental Benefits Status eTable 2. Sensitivity Analyses eTable 3. Robustness Checks [file jamanetwopen-e2124144-s001.pdf]

Supplementary Online Content

Elani HW, Kawachi I, Sommers BD. Dental outcomes after Medicaid insurance coverage expansion under the Affordable Care Act. *JAMA Netw Open*. 2021;4(9):e2124144. doi:10.1001/jamanetworkopen.2021.24144

eTable 1. Classification of States by Medicaid Expansion and State Dental Benefits Status

eTable 2. Sensitivity Analyses

eTable 3. Robustness Checks

© 2021 Elani HW et al. *JAMA Network Open*.

eTable 1. Classification of States by Medicaid Expansion and State Dental Benefits Status

	Medicaid adult dental benefits	
**Medicaid expansion status**	**States with dental benefits**	**States without dental benefits**
**Expansion states**	AK, AR, CA, CO, CT, DC, IA, IL, IN, KY, MA, MI, MN, ND, NJ, NM, NY, OH, OR, PA, RI, VT, WA.	AZ, DE, HI, LA, MD,MT, NH, NV, WV.
**Non expansion states**	NC, NE, SC, SD, WI, WY.	AL, FL, GA, ID, KS, ME, MO, MS, OK, TN, TX, UT, VA.

Source:

1. Kaiser Family Foundation; Status of State Medicaid Expansion Decisions. https://www.kff.org/medicaid/issue-brief/status-of-state-medicaid-expansion-decisions-interactive-map/. Accessed March 13, 2019.

2. Centers for Medicare and Medicaid Services. Medicaid State Plan Amendments. https://www.medicaid.gov/state-resource-center/medicaid-state-plan-amendments/index.html. Accessed March 13, 2019.

3. Center for Health Care Strategies. Medicaid adult dental benefits: An overview. 2019; https://www.chcs.org/resource/medicaid-adult-dental-benefits-overview/. Accessed April 10, 2020.

© 2021 Elani HW et al. *JAMA Network Open*.

eTable 2. Sensitivity Analyses

	Difference-in-differences net change after expansion					
	Excluding pregnant women			Excluding early expansion states		
	%	*P value*	95% CI	%	*P value*	95% CI
**Health coverage**						
**Medicaid coverage**						
Full sample	4.6	0.183	(-2.3 to 11.5)	7.9	0.046	(0.1 to 15.7)
States with dental benefits	8.5	0.029	(0.9 to 16.0)	12.7	0.001	(6.6 to 18.7)
States without dental benefits	1.6	0.841	(-14.9 to 18.1)	2.9	0.715	(-13.4 to 19.2)
**Uninsured status**						
Full sample	-5.8	0.258	(-15.9 to 4.4)	-10.6	0.061	(-21.8, 0.5)
States with dental benefits	-13.1	0.001	(-19.5 to -6.7)	-16.8	0.037	(-32.3 to -1.2)
States without dental benefits	-1.6	0.844	(-17.9 to 14.8)	-4.1	0.577	(-19.2 to 11.0)
**Access to dental care**						
**Dental visit in previous year**						
Full sample	13.6	0.003	(5.0 to 22.1)	13.1	0.061	(-0.7, 26.8)
States with dental benefits	13.0	0.004	(4.8 to 21.2)	13.8	0.045	(0.4 to 27.3)
States without dental benefits	23.6	0.026	(3.2 to 44.0)	21.4	0.047	(0.4 to 42.4)
**Inability to afford dental care in the previous year**						
Full sample	-10.0	0.094	(-21.8 to 1.8)	-16.3	0.012	(-28.7 to -3.9)
States with dental benefits	-18.2	0.001	(-27.8 to -8.6)	-26.7	0.001	(-33.1 to -20.2)
States without dental benefits	-12.7	0.151	(-30.5 to 5.1)	-12.2	0.194	(-31.2 to 6.8)
**Clinically examined dental outcomes**						
**Untreated decayed teeth**						
Full sample	-6.5	0.191	(-16.3 to 3.4)	-8.3	0.295	(-24.3 to 7.7)
States with dental benefits	-18.5	0.001	(-27.3 to -9.7)	-21.9	0.018	(-39.1 to -4.7)
States without dental benefits	-4.0	0.524	(-16.8 to 8.8)	-1.2	0.796	(-11.0 to 8.5)
**Filled teeth**						
Full sample	3.3	0.510	(-6.8 to 13.5)	10.8	0.041	(0.5 to 21.1)
States with dental benefits	12.3	0.059	(-0.5 to 25.2)	22.5	0.001	(12.7 to 32.3)
States without dental benefits	-2.3	0.790	(-20.3 to 15.6)	-0.8	0.924	(-18.4 to 16.7)
**Number of missing teeth**						
Full sample	-0.5	0.461	(-1.7 to 0.8)	-0.1	0.926	(-1.9 to 1.7)
States with dental benefits	-1.3	0.158	(-3.0 to 0.5)	-1.8	0.252	(-5.0 to 1.4)
States without dental benefits	1.2	0.042	(0.0 to 2.4)	1.3	0.056	(0.0 to 2.7)
**Presence of functional dentition**						
Full sample	2.0	0.510	(-4.1 to 8.2)	0.5	0.903	(-8.2 to 9.3)
States with dental benefits	5.8	0.134	(-2.0 to 13.6)	9.3	0.159	(-4.2 to 22.8)
States without dental benefits	-8.6	0.006	(-14.4 to -2.8)	-9.1	0.003	(-14.6 to -3.6)
**Self-reported dental outcomes**						
**Fair or poor oral health**						
Full sample	-2.9	0.424	(-10.1 to 4.3)	-5.0	0.343	(-15.7 to 5.6)
States with dental benefits	-1.5	0.824	(-15.4 to 12.4)	-5.7	0.515	(-24.0 to 12.6)
States without dental benefits	-2.4	0.766	(-18.9 to 14.1)	-2.4	0.752	(-18.2 to 13.4)
**Daily teeth flossing**						
Full sample	-0.2	0.974	(-9.8 to 9.5)	-2.6	0.598	(-12.5 to 7.3)
States with dental benefits	10.3	0.003	(3.9 to 16.7)	7.8	0.028	(1.0 to 14.5)
States without dental benefits	-13.3	0.015	(-23.7 to -2.9)	-12.9	0.009	(-22.2 to -3.6)

Abbreviations: CI= confidence interval.

Note: Study sample limited to adults ages 19 to 64 with income up to 138 percent of the federal poverty level. Models adjusted for age, sex, race, education, marital status, employment, citizenship, state-year unemployment rate, number of dentists per capita in each state-year, and

© 2021 Elani HW et al. *JAMA Network Open.*

state. All analyses used robust standard errors clustered by state. Expansion states can include AK, AR, AZ, CA, CO, CT, DC, DE, HI, IA, IL, IN, KY, LA, MA, MD, MI, MN, MT, ND, NH, NJ, NM, NV, NY,OH,OR ,PA ,RI, VT ,WA, WV. Expansion states that provide dental benefits can include: AK, AR, CA, CO, CT, DC, IA, IL, IN, KY, MA, MI, MN, ND, NJ, NM, NY, OH, OR, PA, RI, VT, WA. Expansion states that don’t provide dental benefits can include: AZ, DE, HI, LA, MD,MT, NH, NV, WV. Nonexpansion states can include: AL, FL, GA, ID, KS, ME ,MO, MS, NC, NE, OK, SC, SD, TN, TX, UT, VA, WI, WY. Nonexpansion states that provide dental benefits can include: NC, NE, SC, SD, WI, WY. Nonexpansion states that don’t provide dental benefits include: AL,FL, GA, ID, KS, ME, MO, MS, OK, TN, TX, UT, VA.

© 2021 Elani HW et al. *JAMA Network Open*.

eTable 3. Robustness Checks

	Placebo Expansion		
	Difference-in-differences net change after expansion		
	%	*P value*	95% CI
**Health coverage**			
**Medicaid coverage**			
Full sample	-4.2	0.288	(-12.2 to 3.7)
States with dental benefits	-9.2	0.060	(-18.8 to 0.4)
States without dental benefits	-2.3	0.455	(-8.9 to 4.2)
**Uninsured status**			
Full sample	11.5	0.006	(3.6 to 19.4)
States with dental benefits	13.2	0.001	(8.4 to 18.1)
States without dental benefits	14.4	0.035	(1.2 to 27.5)
**Access to dental care**			
**Dental visit in previous year**			
Full sample	-17.3	0.001	(-26.4 to -8.2)
States with dental benefits	-1.3	0.880	(-19.7 to 17.1)
States without dental benefits	-22.7	0.009	(-38.7 to -6.7)
**Inability to afford dental care in the previous year**			
Full sample	-8.6	0.208	(-22.4 to 5.1)
States with dental benefits	-14.5	0.032	(-27.5 to -1.5)
States without dental benefits	-9.3	0.432	(-34.0 to 15.4)
**Clinically examined dental outcomes**			
**Untreated decayed teeth**			
Full sample	12.3	0.012	(2.9 to 21.7)
States with dental benefits	5.2	0.182	(-2.8 to 13.2)
States without dental benefits	11.9	0.067	(-0.9 to 24.7)
**Filled teeth**			
Full sample	-14.0	0.023	(-25.9 to -2.1)
States with dental benefits	-16.3	0.011	(-28.0 to -4.5)
States without dental benefits	-6.4	0.169	(-15.9, 3.1)
**Number of missing teeth**			
Full sample	0.7	0.469	(-1.2 to 2.5)
States with dental benefits	0.7	0.468	(-1.3 to 2.8)
States without dental benefits	-0.1	0.922	(-2.6 to 2.4)
**Presence of functional dentition**			
Full sample	-0.8	0.826	(-7.7 to 6.2)
States with dental benefits	1.3	0.779	(-8.1 to 10.6)
States without dental benefits	1.1	0.798	(-7.6 to 9.7)
**Self-reported dental outcomes**			
**Fair or poor oral health**			
Full sample	4.9	0.405	(-6.9 to 16.7)
States with dental benefits	10.3	0.054	(-0.2 to 20.7)
States without dental benefits	1.6	0.821	(-13.3 to 16.5)
**Daily teeth flossing**			
Full sample	-1.1	0.847	(-12.9 to 10.6)
States with dental benefits	-4.7	0.367	(-15.5 to 6.0)
States without dental benefits	6.0	0.540	(-14.5 to 26.5)

Abbreviations: CI= confidence interval.
Note: Study sample limited to adults ages 19 to 64 with income up to138 percent of the federal poverty level. Models adjusted for age, sex, race,

© 2021 Elani HW et al. *JAMA Network Open*.

education, marital status, employment, citizenship, state-year unemployment rate, number of dentists per capita in each state-year, and state. All analyses used robust standard errors clustered by state. Placebo expansion using NHANES data from 2009 to 2013 and year 2012 as the ACA implementation year. Expansion states can include AK, AR, AZ, CA, CO, CT, DC, DE, HI, IA, IL, IN, KY, LA, MA, MD, MI, MN, MT, ND, NH, NJ, NM, NV, NY,OH,OR ,PA ,RI, VT ,WA, WV. Expansion states that provide dental benefits can include: AK, AR, CA, CO, CT, DC, IA, IL, IN, KY, MA, MI, MN, ND, NJ, NM, NY, OH, OR, PA, RI, VT, WA. Expansion states that don’t provide dental benefits can include: AZ, DE, HI, LA, MD,MT, NH, NV, WV. Nonexpansion states can include: AL, FL, GA, ID, KS, ME ,MO, MS, NC, NE, OK, SC, SD, TN, TX, UT, VA, WI, WY. Nonexpansion states that provide dental benefits can include: NC, NE, SC, SD, WI, WY. Nonexpansion states that don’t provide dental benefits include: AL,FL, GA, ID, KS, ME, MO, MS, OK, TN, TX, UT, VA.

© 2021 Elani HW et al. *JAMA Network Open*.
